# The utility of artificial intelligence in visualization of pediatric gastrointestinal mucosa

**DOI:** 10.3389/fped.2025.1739000

**Published:** 2026-01-12

**Authors:** Jeremy W. Stewart, Bradley A. Barth, Isabel Rojas

**Affiliations:** Division of Pediatric Gastroenterology, Hepatology and Nutrition, University of Texas Southwestern Medical Center, Dallas, TX, United States

**Keywords:** artificial intelligence, deep learning, gastroenterology, inflammatory bowel disease, machine learning, pediatric gastroenterology, video capsule endoscopy

## Abstract

The utilization of artificial intelligence (AI) is rapidly expanding in all areas of medicine. Pediatric gastroenterology is among the fields exploring the use of AI to better visualize the gastrointestinal tract and improve diagnosis, disease subtyping, lesion detection, risk prediction, and treatment optimization for better patient outcomes. AI shows promising developments and applications in complex diseases, such as Crohn's disease, polyposis syndromes, and eosinophilic esophagitis, where diagnosis and initial or subsequent management are impacted by mucosal visualization and analysis. This article summarizes how AI, machine learning, and these complex networks work in addition to addressing the limitations and ethical challenges faced with use of this budding technology. Although most available information on this topic comes from adult literature, this discussion focuses on current and emerging pediatric research and applications of AI in pediatric diagnostic and interventional endoscopy.

## 1 Introduction

Artificial intelligence (AI) is increasingly integrated into daily life, improving efficiency, creativity, and productivity, from generating art and music to drafting documents and performing complex calculations. Healthcare has rapidly adopted AI, with an exponential rise in PubMed-indexed publications over the past 20–30 years. Between 2020 and 2025, over 150,000 articles included the terms “artificial intelligence, “deep learning,” or “machine learning,” of which 3,142 pertain to gastroenterology and only 220 specifically address pediatric populations (1).

Recently, generative AI (GAI) has emerged as a clinical tool capable of answering medical queries, assisting with documentation, and generating patient-facing materials (2). AI applications show promise in complex gastrointestinal diseases such as Crohn's disease, polyposis syndromes, and eosinophilic esophagitis, where diagnosis and management rely heavily on mucosal visualization and analysis. As in most emerging medical technologies, pediatric research lags behind adult studies. Pediatric gastroenterology, however, presents unique opportunities to apply AI and GAI in both clinical care and research. This article provides an overview of AI, GAI, and machine learning principles, and reviews current literature, limitations, and ethical challenges related to their use in visualizing the pediatric gastrointestinal tract.

## 2 How it works

A basic understanding of AI, its terminology and processes is essential. The terms artificial intelligence, generative AI, machine learning, and deep learning are often used interchangeably but represent distinct concepts.

Artificial intelligence broadly refers to the simulation of human intelligence by a system or a machine (3, 4). First coined by John McCarthy at a Dartmouth College conference in 1956, AI was initially developed to prove mathematical theorems and solve algebraic problems. AI requires perceptual, cognitive, and decision-making capabilities, traditionally assessed by the “Turing Test,” which evaluates whether a machine's responses are indistinguishable from a human's (5). According to Xu et al, AI development involves data collection, computing power, an AI framework, and machine learning. Generative AI refers to systems that generate new content after training on large datasets (4). Examples include creating an educational animation or video explaining endoscopy (6).

Machine learning (ML), a subset of AI, recognizes data patterns through algorithms such as regression models and decision trees. A generic form of ML is supervised learning; it uses labeled data with adjustable parameters to reach desired outputs, whereas unsupervised learning identifies features without labeled input. Deep learning (DL), a subset of ML, employs multilayered neural networks, e.g., convolutional neural networks (CNN), to create models for more complex pattern recognition, mimicking the human nervous system. Unlike ML, DL models “learn” from data through hierarchical pattern recognition and representation learning (7). Examples of ML include email filters or electronic medical record medications alerts, while DL applications include image or speech recognition. Understanding these distinctions is critical to applying AI effectively.

## 3 Visualization of the GI tract

As in other medical fields, AI shows promise in enhancing diagnostic precision and treatment accuracy through improved visualization of the gastrointestinal tract. A 2021 systematic review assessed AI and predictive models for detecting malignant and non-malignant lesions in the upper and lower GI tract, hepatobiliary system, and pancreas (8). A meta-analysis of 43 studies involving 15,000 tandem colonoscopies reported miss rates of 26% for adenomas, 9% for advanced adenomas, and 27% for serrated polyps, underscoring the potential of AI to reduce missed lesions (9).

The “Computer-aided detection” (CADe) systems aim to improve real-time identification of polyps and malignancies during colonoscopy. One CADe model, trained on 69,000 chromoendoscopy and narrow band imaging (NBI) images, achieved accuracies of 98% and 96%, respectively, exceeding expert and nonexpert endoscopists (10). CADe systems demonstrate high sensitivity but depend on adequate bowel preparation. Table 1 lists currently FDA-approved CADe and other AI systems in the United States for gastroenterology (11–18).

**Table 1:** This table includes the currently FDA approved systems for lesion detection available in the United States with the corresponding company and/or manufacturer.

Device/system	Company/manufacturer	FDA clearance date
Colonoscopy systems		
GI Genius™	Cosmo Artificial Intelligence	July 2021
EndoScreener®	Chengdu Wision Medical Device Co	November 2021
SKOUT®	Iterative Health/Provation	August 2022
MAGENTIQ-COLO™/ME-APDS™	Magentiq Eye	July 2023
ColonPRO™	Cosmo Artificial Intelligence	January 2024
CAD EYE®	FujiFilm	March 2024
CADDIE™	Odin Medical Limited/Olympus	July 2024
Video capsule endoscopy system		
NaviCam ProScan—assisted reading tool	Ankon Technologies	December 2023

While there are more systems and versions of the software available outside the United States, the systems are not yet FDA approved.

In contrast, “computer-aided diagnosis” (CADx) systems classify detected abnormalities to guide management decisions, such as distinguishing neoplastic from non-neoplastic lesions (19). Limitations include scarce external validation and the absence of FDA- approved systems in the United States.

Although no CADe or CADx models have been studied specifically in pediatrics, their application could benefit polyposis disorders such as juvenile polyposis syndrome, Peutz-Jeghers syndrome, and familial adenomatous polyposis, where routine endoscopic surveillance is essential (20–22). Broader adoption would require multicenter data collection and pediatric-specific model training.

### 3.1 Video capsule endoscopy

Since its FDA approval in 2001, video capsule endoscopy (VCE) has provided a less invasive method to visualize the gastrointestinal tract. Indications include small bowel bleeding, Crohn's disease activity, surveillance of polyposis syndromes, and evaluation of malabsorption disorders such as Celiac disease (23). The capsule, swallowed or placed endoscopically captures 2–35 images per second over 8–10 h, creating a substantial time burden for gastroenterologists and making it an ideal target for AI-assisted analysis (23, 24). In a multicenter trial of 137 patients across 13 European centers, the Navicam SB system using ProScan (a deep neural network-based AI system) reduced average reading time from 33.7 to 3.8 min and improved lesion detection from 62.4% to 73.7%. Kroner et al. also reviewed AI use in VCE since early ML development, and the review summarizes findings from 31 studies of GI bleeding, angioectasia, small intestinal ulcers, celiac disease, and even Hookworm infection (8, 25).

Ding et al. developed a DL model from over 100 million images from VCE, to identify and categorize findings, achieving 99.9% sensitivity and 99.88% specificity, compared with gastroenterologists’ 74.6% and 76.9% sensitivities for “per-patient” and “per-lesion” analyses, respectively (24). Another striking finding from this study includes the reading time of the CNN model of 5.9 min when compared to 96.6 min of conventional reading (24). Another multicenter CNN study analyzed 66,208 images using RAPID reader software QuickView mode, identifying 44,684 abnormalities, mucosal breaks, angioectasia, protruding lesions, and blood content in alignment with CEST standardized reporting terminology, with detection rates of 95.7%, 75.9%, 98.8%, and 100% respectively, outperforming QuickView (99% vs. 89%) (26, 27). Its adaptive sampling rate allowed more efficient image review. Additional AI-based VCE studies have combined endoscopic, histologic, MRI, and genetic data for IBD detection in adults.

While pediatric comparative studies are lacking, small bowel findings are generally similar between adults and children. Huang et al. retrospectively analyzed VCE from 162 pediatric patients using four DL models (DenseNet121, Visual Geometry Group-16, ResNet50, and Vision Transformer), with DensNet121 and Resnet50 achieving 90.6% and 90.5% accuracy, respectively (28). This remains the only pediatric-specific VCE AI published study to date, highlighting the need for larger pediatric datasets to enhance accuracy and generalizability.

### 3.2 Inflammatory bowel disease

Inflammatory bowel disease (IBD) can involve the entire gastrointestinal tract and requires accurate histologic, clinical, and endoscopic assessment for diagnosis and staging. AI-assisted technologies have shown potential benefits in imaging, biomarker evaluation, and treatment decisions (29–31). Endoscopic imaging has also been used to develop DL models for evaluating and grading inflammatory bowel disease (IBD) severity. Because grading systems are subject to inter- and intra-observer variability, AI-assisted technology can help standardize assessments. A DL model for ulcerative colitis (UC) severity grading demonstrated sensitivity, specificity, and accuracy comparable to experienced human reviewers (32). Another study developed a multilayered CNN that analyzed 16,514 colonoscopic images from 3,082 patients to distinguish remission (Mayo score 0 or 1) from moderate to severe disease (Mayo score 2 or 3), achieving 83% sensitivity and 96% specificity (33). A meta-analysis of 12 studies evaluating deep ML and CNN algorithms found excellent accuracy, sensitivity, and specificity for UC severity scoring, with the Ulcerative Colitis Endoscopic Index of Severity (UCEIS) outperforming the Mayo Endoscopic Score (MES) (34).

Although multiple adult studies support AI-assisted endoscopic visualization in IBD, similar pediatric studies are lacking. This gap highlights the need for pediatric-specific AI models, particularly given the distinct features and presentation patterns of pediatric and very-early-onset IBD (35).

### 3.3 Celiac disease

The use of DL and ML in Celiac disease has been explored by comparing expert human readers with machine learning algorithm diagnosis and disease activity monitoring of VCE studies of 63 adult patients. The study found strong agreement between the two groups in identifying villous damage (36). Other DL models have aimed to improve lesion detection accuracy. For example, Ciaccio et al. Developed a “color masking” technique to filter extraneous image features, achieving 80% accuracy in distinguishing villous atrophy compared to normal controls (37). Although pediatric-focused studies are limited, Syed et al. Developed a CNN to differentiate pathological vs. healthy duodenal tissue in 102 children across multiple institutions, reporting a 93.4% detection accuracy (38). These findings highlight the promise of AI-based tools for enhancing diagnostic precision in both adult and pediatric celiac disease, warranting further validation in larger pediatric cohorts.

### 3.4 Other uses of AI in gastroenterology

In another adult study, a DL algorithm-based diagnostic system, HOPE AI, was developed using 308,887 endoscopic images and 197 videos from 6,207 patients to detect *Helicobacter pylori* infection. The model demonstrated higher sensitivity than senior endoscopists (85.7% vs. 68%), illustrating the potential of AI to enhance diagnostic accuracy. The study also incorporated external geographic validation across multiple centers and prospectively enrolled patients in later phases, strengthening its generalizability (39).

## 4 Medical society guidelines

Recognizing the growing role of AI in gastroenterology, several major societies have recently issued guidance. In January 2025, the American Society for Gastrointestinal Endoscopy (ASGE) AI Task Force released consensus statements outlining the current landscape of AI applications, developed by experts in endoscopy, technology, regulatory authorities, and other subspecialties (40). These statements emphasized AI's potential to enhance lesion detection and characterization, data quality and modeling accuracy, diagnostic precision, prognostication, clinical research, and medical education. They also encourage collaboration among engineers, researchers, and gastroenterologists to advance understanding and responsible integration of AI.

In April 2025, the American Gastroenterology Association (AGA) published a living clinical practice guideline on CADe systems, issuing no recommendation for or against their use due to the “close tradeoff between desirable and undesirable effects” and current evidence limitations (41). Similarly, the European Society of Gastrointestinal Endoscopy (ESGE) released a position statement comparing AI to experienced endoscopists, addressing applications such as landmark recognition and completeness in upper GI endoscopy, detection and resection of neoplastic or cancerous lesions, automated reading of small bowel capsule studies, and characterization (CADx) of polyps of ≤5 mm and selection for resection in ≥6 mm (42).

To date, the North American Society for Pediatric Gastroenterology, Hepatology & Nutrition (NASPGHAN) has not published position statements or guidelines on the use of AI in pediatric gastroenterology.

## 5 Teaching

Artificial intelligence is already influencing the education and training of adult and pediatric gastroenterologist fellows, much like other medical specialties impacted by its integration. Although no studies specifically address its effect on pediatric trainees, Kang et al. reviewed the potential benefits, challenges, and limitations AI presents to medical educators and learners. A key point from this review emphasized that the question is not *if* AI will be used, but *how* it should be applied (43). While there are several studies citing improved detection rates of lesions with trainee utilization of AI detection in VCE (44) and reduced reading time (45), misidentification of lesions as neoplastic with increase in unnecessary biopsies or interventions, and thus risk for harm, were noted with the CADe system (10); however, the CADx system previously discussed is a potential tool for trainees in an added layer of diagnostic assessments (46, 47). One concern in medical education (and other areas of teaching) is the potential overreliance on technology at the expense of experiential learning. The impact of AI on trainee education, particularly in pediatric gastroenterology, warrants further exploration to ensure that educational quality and clinical judgement are preserved for the next generation of physicians.

## 6 Limitations

While AI has certainly proven a valuable technological advance, several limitations remain in its application to gastroenterology imaging. A recurring theme throughout this manuscript is the limited pediatric data and the need for caution when extrapolating adult findings to children. A manual review of the pediatric radiology literature involving AI and machine learning identified small dataset size as the most significant limitation, followed by lack of external validation and other methodological constraints (48). Small sample sizes and preclinical studies hinder robust dataset generation; however, CADe remains one of the most extensively evaluated AI technologies within gastroenterology (49, 50).

A limited number of studies also complicates differentiation between IBD and mimicking pathologies, such as Behçet's disease, gastrointestinal tuberculosis, ischemic colitis, infectious colitis, and distinguishing Crohn's disease from Ulcerative Colitis (51–55). One study evaluating eye-tracking metrics and polyp detection with CADe systems found similar reaction times but increased misinterpretation of normal mucosa, underscoring the importance of maintaining physician oversight rather than deferring to automated systems (56).

Cost-effectiveness is another consideration. Although economic analyses are limited, one microsimulation study estimated AI-assisted colon cancer screening could save $57 per individual screened, an approximately $290 million if applied at the U.S. population level (57). Finally, automation bias is a concern for physicians where the output of an AI system is chosen over the physician's own decisions, even if incorrect; conversely, automation aversion can develop when a physician may not trust the output even if it is correct (50, 58). Additional limitations are evident when considering the ethical and regulatory factors discussed in the following section. Ultimately, AI systems, models, and networks need larger, multicenter studies with external validation before widespread use.

## 7 Ethics

As gastroenterology specialists explore the use of AI to enhance patient care, practice efficiency, cost-effectiveness, and reduce administrative load, it is essential to recognize the accompanying ethical challenges. Responsible implementation requires maintaining safety, transparency, and ethical integrity. While ethical considerations in AI warrant extensive discussion, this section provides only a brief overview.

Artificial intelligence introduces multiple ethical concerns, including lack of transparency and reliability, potential for bias, data security and confidentiality, inequity, potential for “hallucinations,”, and environmental impact (59, 60). Because system training depends on real patient data, ensuring privacy and compliance with the Health Insurance Portability Accountability Act of 1996 (HIPAA) remains a significant challenge (61). Although training data should be de-identified, the risk of “re-identification” persists, especially in small or rare pediatric datasets where case uniqueness may inadvertently reveal patient identity (62). Bias may arise when training data do not represent the true population, potentially perpetuating inequities in diagnosis or treatment (63, 64). Transparency is undoubtably one of the challenging aspects of AI, the so-called “black box” problem, where the inner workings of complex algorithms are not easily explainable. Proposed strategies to mitigate this issue include transparency of data sources, algorithms, processes, and outcomes, allowing users to interpret and validate results (65, 66).

AI “hallucinations”, a phenomenon seen in large language models, further complicate trust and reliability. These models can generate inaccurate, unreliable diagnostic and therapeutic data from flawed reasoning pathways (67). For instance, a generative AI tool might produce an image of a physician with anatomically inconsistent details, an example of nonsensical but confident output. Mitigation strategies include quantifying hallucination frequency and enforcing continuous oversight and stewardship of large language models (68).

Regulatory efforts toward the use of AI are at a pivotal point as we see a dramatic incorporation of its use in everyday life and in healthcare. Currently the FDA, European Union (EU), World Health Organization (WHO), and United Kingdom (UK) are among the many governing bodies with processes for approval of new or emerging AI technology with attention to the training data, performance, bias, post-marketing plans, changes (if added to an existing approved technology), monitoring, transparency, and safety, among others (69–74).

## 8 Future direction

The future for AI in pediatric GI is likely to mirror the current research in adults. With reduction in reading time reported by VCE, these robust studies will likely continue to improve datasets and thus the efficiency of the models developed (75). Cloud-based AI detection software is also likely to continue to develop like the OLYSENSE CAD/AI, which has approval by the FDA for CADDIE to assist in detecting (without diagnosing) colorectal polyps (17), but the software has support for SMARTIBD to aid in analyzing ulcerative colitis during colonoscopy (76). Models that combine different aspects of diagnoses, like histological findings with visual findings, particularly in IBD, are also expected to continue to develop. Vision Transformer (ViT) architecture is another emerging technology that evaluates images across multiple models and networks to capture the relationships of complex datasets. ViT or a combination of ViT-CNN could be used to detect and filter image imperfections, such as poor lighting, debris, bubbles or poor prep (77, 78). Utilizing predetermined change control plans (PCCPs), the FDA issued guidance to support a simplified approval pipeline to allow systems to be updated without a new device approval (79).

## 9 Discussion

Artificial intelligence technologies, including machine learning, deep learning, convolutional neural networks, large language models, and generative AI, will continue to shape healthcare. These models and systems show promise of reducing errors or missed lesions, as in CADe/CADx systems, reduce time spent viewing thousands of images in VCE, better characterize chronic diseases to help create precision medicine in IBD and other diseases of the GI tract, and have potential to help improve efficiency to minimize additional cognitive load. As we recognize the roles AI can play in the care of patients, it is evident that more pediatric data across more centers is needed to create robust and accurate dataset training to prevent bias, among other challenges, to develop better models for Pediatric GI. Being at the forefront of the development of these models and systems, we have a duty to our trainees to not only prevent reliance on AI for patient care but also to instill that AI is best used to assist and augment our current care without replacing our knowledge and skills. We should strive to maintain a boundary of assistance from the tools while stressing the importance of validation and approval of the information, algorithm, or other output generated. Clinicians must also strive to continue preserve patient autonomy, beneficence, non-maleficence, and maintain justice in the ethical use of this developing and emerging technology.
